# Serum Levels of Asprosin, a Novel Adipokine, Are Significantly Lowered in Patients with Acromegaly

**DOI:** 10.1155/2020/8855996

**Published:** 2020-12-14

**Authors:** Xiaoan Ke, Lian Duan, Fengying Gong, Yuelun Zhang, Kan Deng, Yong Yao, Linjie Wang, Hui Pan, Huijuan Zhu

**Affiliations:** ^1^Department of Endocrinology, State Key Laboratory of Complex Severe and Rare Diseases, Key Laboratory of Endocrinology of National Health Commission, Peking Union Medical College Hospital, Chinese Academy of Medical Science and Peking Union Medical College, Beijing 100730, China; ^2^Central Research Laboratory, Peking Union Medical College Hospital, Chinese Academy of Medical Science and Peking Union Medical College, Beijing 100730, China; ^3^Department of Neurosurgery, Peking Union Medical College Hospital, Chinese Academy of Medical Science and Peking Union Medical College, Beijing 100730, China

## Abstract

**Background:**

Asprosin is a novel identified adipokine secreted mainly by white adipose tissue, which is elevated in metabolic diseases such as diabetes and obesity. Acromegaly is a syndrome caused by pituitary growth hormone (GH) cell adenoma with excessive GH secretion. Serum adipocytokines levels may be involved in abnormal glycolipid metabolism in acromegaly patients.

**Objectives:**

To investigate serum asprosin levels in acromegaly patients and its correlation with high GH levels and glucolipid metabolic parameters.

**Methods:**

A retrospective case-control study was conducted and 68 acromegaly patients and 121 controls were included in this study. Clinical information and laboratory examinations were collected and serum asprosin levels were measured by commercial ELISA kits.

**Results:**

Serum asprosin levels in acromegaly patients were significantly lower than controls (*P* < 0.001). Serum asprosin levels in patients with the course of acromegaly ≥5 years (compared with <5 years), high area under curve of growth hormone (GH-AUC) after 75 g oral glucose tolerance test (OGTT) (compared with low GH-AUC patients), and high IGF-1 SDS group (compared with low IGF-1 SDS group) were significantly reduced (all *P* < 0.05). Serum asprosin levels in acromegaly patients were negatively correlated with the course of acromegaly, IGF-1 SDS, nadir growth hormone value (GH-Nadir), and GH-AUC after OGTT. Multiple stepwise linear regression indicated that acromegaly was an independent influencing factor of serum asprosin levels. According to serum asprosin levels tertiles, the risk of acromegaly in the lowest group was 2.67 times higher than the highest group (OR ＝ 3.665, 95% CI 1.677 ∼ 8.007, *P*=0.001), and the increased risk of the lowest group still existed after adjusting for gender, age, BMI, and TC (Model 2).

**Conclusions:**

Serum asprosin levels in acromegaly patients are lowered, which may be related to increased blood glucose and reduced body fat mass caused by long-term high GH levels exposure.

## 1. Introduction

For many years, adipose tissue has been regarded as the passive energy store organ. In recent years, adipose tissue has been thought to be the largest active endocrine organ in the human body, which can synthesize and secrete a variety of adipokines and participate in glucolipid metabolism through endocrine, autocrine, and paracrine pathways [1]. In 2016, Romere et al. [2] discovered asprosin as a new adipokine by sequencing and mechanically exploring neonatal premature aging syndrome patients with ***FBN1*** mutations. Asprosin is mainly expressed and secreted by white adipose tissue, which can break down liver glycogen and raise blood glucose levels. Pathologically elevated asprosin levels also are observed in insulin-resistant patients and mice. Besides, asprosin leads to islet *β*-cell secretion dysfunction and apoptosis [3] and reduces skeletal muscle insulin sensitivity [4] through inflammatory pathways. Recently, Li et al. [5] have found that asprosin mediates liver glycogenolysis by binding to the liver OLFR734 receptor using siRNA interference technology and gene knockout animal model. In addition, asprosin is also an anorectic hormone [6]. The latest research performed by Liu et al. [7] have revealed that asprosin binds to the central OLFR734 receptor and promotes appetite by enhancing olfaction and activating AgRP neuron. Moreover, many clinical studies have observed that serum asprosin levels are increased in patients with metabolic diseases such as obesity, type 2 diabetes, and polycystic ovary syndrome (PCOS) [8–11]. Therefore, asprosin may be a potential biomarker for abnormal glucolipid metabolism related diseases [11].

Acromegaly is a syndrome characterized by excessive growth hormone (GH) secretion, usually caused by pituitary growth hormone cell adenoma. Patients with acromegaly have been in a state of high GH levels. As an important metabolic regulating hormone, growth hormone can promote protein synthesis and lipolysis. Therefore, patients with acromegaly usually show reduced fat mass and increased lean body mass. Patients with acromegaly often have abnormal glucolipid metabolism, including insulin resistance and diabetes, which may be related to high GH status, resulting in adipose tissue dysfunction and the imbalance of adipocytokines synthesis and secretion [12]. Several studies have reported serum levels of different adipokines in acromegaly patients and the correlations between adipokines and glucolipid metabolism indexes. Silha et al. [13] and Bolanowski et al. [14] revealed that serum leptin levels decreased compared to controls in acromegaly patients. Fukuda et al. [15] showed that, compared with controls, serum adiponectin levels decreased (4.3 ± 1.8 vs 6.7 ± 1.8 g/ml, *P* < 0.001) in acromegaly patients, but another study by Ronchi et al. showed no serum adiponectin levels change was observed in acromegaly patients [16]. In general, serum leptin levels in patients with acromegaly are reduced, while other adipocytokines have been assessed in a few studies with inconsistent conclusions [17].

As a novel identified adipocytokine recently, asprosin has been considered to be a potential biomarker for abnormal glucolipid metabolic disorders such as obesity and type 2 diabetes. However, serum asprosin levels and its association with glucolipid metabolism parameters in acromegaly patients have not been investigated and as far as we know, there is no relevant literature. Therefore, this study collected clinical data and laboratory examinations of acromegaly patients, measured their serum asprosin levels by ELASA, and investigated serum asprosin levels in acromegaly patients, as well as its correlation with high GH levels and glycolipid metabolism parameters.

## 2. Subjects and Methods

### 2.1. Subjects

A total of 68 patients with acromegaly were enrolled due to pituitary growth hormone cell adenoma undergoing neurosurgery in Peking Union Medical College Hospital from October 2012 to May 2013. There were 21 previously diagnosed acromegaly patients with 13 receiving somatostatin analogue treatment and 1 receiving bromocriptine treatment. 1 of 68 acromegaly patients had been treated with drugs for lowering lipid (Lipitor), but the specific dosage was unknown and the blood lipid data (LDL-C, HDL-C, TC, and TG) were not available and there were 11 acromegaly patients having received the therapy of diabetes. A total of 121 non-acromegaly controls matched with gender, age, and BMI were included from the physical examination center of Peking Union Medical College during the same period. This study was approved by the Ethics Committee of the Peking Union Medical College Hospital, Chinese Academy of Medical Sciences (ethics number: SJ-1233). All research subjects signed informed consent before the study.

### 2.2. Diagnostic Criteria

Diagnostic criteria for acromegaly [18]: clinical manifestations such as special face, the nadir growth hormone value after 75 g oral glucose tolerance test (OGTT) ＞1 ng/ml, or postoperative pathological diagnosis of pituitary growth hormone adenoma. Diagnostic criteria for diabetes based on WHO (1999) standard: impaired fasting blood glucose (IFG): 6.1 mmol/l ≤fasting blood glucose <7.0 mmol/l and two-hour postprandial blood glucose <7.8 mmol/l; impaired glucose tolerance (IGT): 7.8 mmol/l ≤two-hour postprandial blood glucose <11.1 mmol/l and fasting blood glucose <7.0 mmol/l; diabetes (DM): fasting blood glucose ≥7.0 mmol/l and/or two-hour postprandial blood glucose ≥11.1 mmol/l; previously diagnosed with diabetes. Diagnostic criteria for hyperlipidemia [19]: total cholesterol (TC) ≥5.2 mmol/l (200 mg/dl) and/or triglyceride (TG) ≥1.7 mmol/l (150 mg/dl); previously diagnosed with hyperlipidemia. Diagnostic criteria for overweight and obesity were in accordance with the 2003 Guidelines for the Prevention and Control of Overweight and Obesity in Chinese Adults (Trial) [20]: overweight: 24 kg/m^2^ ≤BMI <28 kg/m^2^; obesity: BMI ≥28 kg/m^2^; previously diagnosed with obesity. Diagnostic criteria for hypertension [21]: systolic blood pressure ≥140 mmHg and/or diastolic blood pressure ≥90 mmHg; previously diagnosed with hypertension.

### 2.3. Clinical Data Collection

Clinical information for preoperative evaluation patients with acromegaly was collected, including general data (sex, age, BMI, blood pressure), chief complaints, current medical history, past medical history, family history, vital signs, physical examination, laboratory examinations, imaging data, etc. Laboratory examinations included alanine aminotransferase (ALT), aspartate aminotransferase (AST), blood creatinine (Cr), urea (UA), total cholesterol (TC), triglyceride (TG), high-density lipoprotein cholesterol (HDL-C), low-density lipoprotein cholesterol (LDL-C), blood glucose values, and growth hormone values after 75 g OGTT (5 samples for 0 minutes, 30 minutes, 60 minutes, 120 minutes, and 180 minutes; GH-AUC and Glu-AUC were calculated using GraphPad Prism 8.0.2). The following clinical information of controls was collected: gender, age, BMI, blood pressure, laboratory examinations, including ALT, AST, Cr, UA, TC, TG, HDL-C, LDL-C, and blood glucose values after OGTT.

### 2.4. Serum Asprosin Measurements

The serum of patients was collected from all subjects in the morning with an empty stomach before surgery in our hospital. 3-4 ml peripheral blood was collected and centrifuged at 3000 r for 10 minutes and then stored in a refrigerator at −80°C. Serum asprosin levels were measured by ELISA kits (USCN Life Science Inc., Wuhan, China, Article no. SEA332Hu) according to the instructions in March 2018. The inter-assay and intra-assay coefficients of variation (CVs) were 12.9% and 6.7%, respectively.

### 2.5. Statistical Analysis

All data were analyzed using SPSS 25.0 statistical analysis, and all graphs were drawn using GraphPad Prism 8.0.2. The Shapiro–Wilk test and the P-P chart were used to test the normality of data, and some non-normal distributed data were analyzed after ln conversion. The independent sample *t* test and non-parametric Mann–Whitney *U* test were used to compare all variables between acromegaly patients and controls. Chi-square test was used to compare the proportions of gender and metabolic diseases between acromegaly patients and controls. Spearman bivariate correlation analysis was used to explore the correlation between serum asprosin levels and other variables. The Spearman correlation coefficient determined the statistical significance level. Kruskal–Wallis H test and one-way analysis of variance were used to compare serum asprosin levels among multiple groups. Multiple stepwise linear regression was used to explore the influencing factors of serum asprosin levels, with serum asprosin levels as dependent variable, group (acromegaly assigned a value of 1 and controls assigned a value of 0), gender (male assigned a value of 1 and female assigned a value of 0), age, BMI, systolic blood pressure (SBP), diastolic blood pressure (DBP), TC, TG, LDL-C, HDL-C, fasting blood glucose (FBG), two-hour postprandial blood glucose (2h-Glu), ALT, AST, Cr, and UA as independent variables. Binary logistic regression was used to analyze the risk of acromegaly according to serum asprosin levels tertiles.

## 3. Results

### 3.1. Basic Characteristics in Acromegaly Patients and Controls

The basic characteristics of acromegaly patients are shown in Table 1. The ratio of male to female in acromegaly patients was 74% (29/39), the average age was 41.3 ± 15.0 years, and the average BMI was 25.79 ± 3.52 kg/m^2^. In these patients, the median nadir GH (GH-Nadir) after OGTT was 10.7 ng/ml, the median area under curve of GH (GH-AUC) after OGTT was 2391.0 ng/ml, and the average IGF-1 SDS was 6.56 ± 2.43. There was no significant difference in sex, age, BMI, and blood pressure between acromegaly patients and controls, suggesting basic characteristics of two groups were consistent.

Compared with controls, patients with acromegaly had higher fasting blood glucose (6.0 ± 1.7 vs 5.4 ± 0.9, *P*=0.003), but lower TC (4.49 ± 0.85 vs 4.81 ± 0.85, *P*=0.033), HDL-C (1.19 ± 0.31 vs 1.40 ± 0.36, *P*=0.004), and LDL-C (2.62 ± 0.74 vs 2.93 ± 0.70, *P*=0.013). In addition, ALT, AST, and Cr in acromegaly patients were all lower than controls.

The occurrence of metabolic diseases in acromegaly patients is shown in Table 2. There were 19 acromegaly patients with diabetes (28.8%), among which 2 patients were treated with insulin and 9 patients were treated with drugs. 7 patients in controls had diabetes (10.6%). The incidence of diabetes in patients with acromegaly was significantly higher than controls (28.8% vs 10.6%, *P*=0.009). There was no statistical difference in the incidence of hypertension, hyperlipidemia, and obesity between two groups.

### 3.2. Serum Asprosin Levels in Acromegaly Patients and Controls

**Figure 1:**
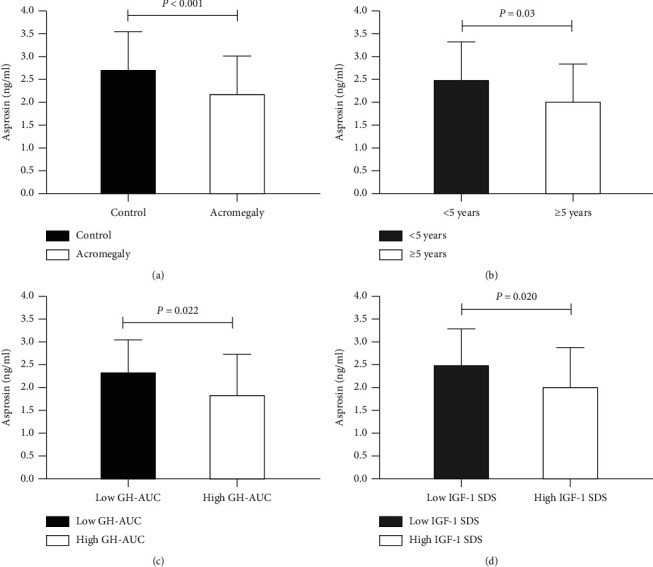
Serum asprosin levels in acromegaly patients. (a) Subjects including acromegaly and controls; (b) acromegaly patients with course ≥5 years and <5 years; (c) acromegaly patients with high GH-AUC and low GH-AUC; (d) acromegaly patients with high IGF-1 SDS and low IGF-1 SDS.

As shown in Figure 1(a), serum asprosin levels in patients with acromegaly were significantly lower than controls (2.18 ± 0.86 vs 2.71 ± 0.86 ng/ml, *P* < 0.001). After grouping the patients in terms of the course of acromegaly, serum asprosin levels in patients with the course of acromegaly ≥5 years were significantly lower in comparison with those with the course of acromegaly <5 years as shown in Figure 1(b) (1.97 ± 0.84 vs 2.44 ± 0.83 ng/ml, *P*=0.030). To further explore the association between serum asprosin levels and serum GH levels, patients were divided into high GH-AUC group (GH-AUC ≥2391 ng/ml) and low GH-AUC group (GH-AUC <2391 ng/ml). Compared to patients with low GH-AUC, serum asprosin levels in patients with high GH-AUC were significantly reduced (1.94 ± 0.91 vs 2.44 ± 0.76 ng/ml, *P*=0.022, Figure 1(c)). In addition, compared with low IGF-1 SDS group (IGF-1 SDS <6.56), serum asprosin levels in high IGF-1 SDS group (IGF-1 SDS ≥6.56) were significantly decreased (1.94 ± 0.86 vs 2.42 ± 0.80 ng/ml, *P*=0.020, Figure 1(d)). Notably, serum asprosin levels in patients with high GH-Nadir (≥10.7 ng/ml) were lower (1.98 ± 0.97 vs 2.40 ± 0.71 ng/ml) than the low GH-Nadir group (<10.7 ng/ml), though there was no statistical difference in serum asprosin levels between two groups (data was not shown).

### 3.3. Subgroups Analysis of Serum Asprosin Levels in Acromegaly

**Figure 2:**
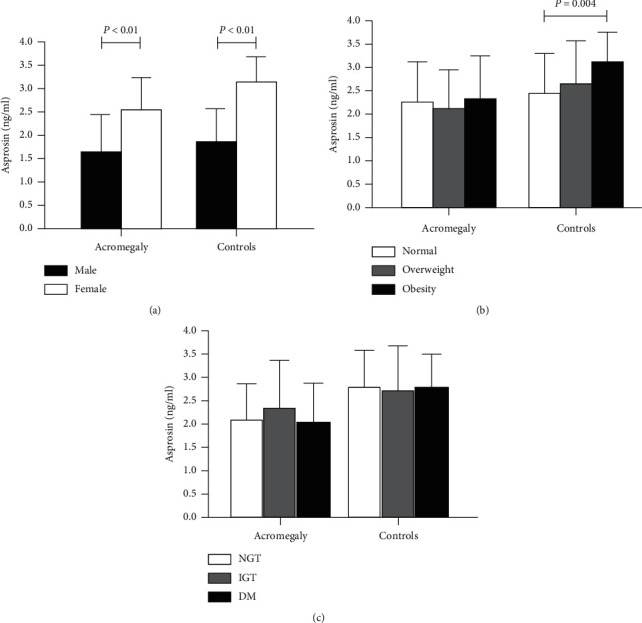
Subgroups analysis of asprosin levels in acromegaly and controls. (a) Male and female in acromegaly and controls; (b) normal, overweight, and obesity in acromegaly and controls; (c) normal glucose tolerance (NGT), impaired glucose tolerance (IGT), and diabetes (DM) in acromegaly and controls.

Next, serum asprosin levels in different subgroups of acromegaly and controls were explored. As shown in Figure 2(a), serum asprosin levels of women were significantly higher than men in acromegaly patients, as well as controls (2.56 ± 0.69 vs 1.66 ± 0.80 ng/ml and 3.15 ± 0.54 vs 1.87 ± 0.71 ng/ml, both *P* < 0.001). There was no difference in serum asprosin levels in acromegaly patients stratified into subgroups by BMI (*H* ＝ 0.834, *P*=0.659, Figure 2(b)). However, serum asprosin levels were different among subgroups in controls (*H* ＝ 10.852, *P*=0.04). After further post hoc pairwise comparisons using Bonferroni correction, serum asprosin levels in obesity were significantly higher than normal in controls (3.08 ± 0.64 ng/ml vs 2.42 ± 0.86 ng/ml, *P*=0.004).

Both acromegaly patients and controls were divided into diabetes (DM), abnormal glucose tolerance (IGT), and normal glucose tolerance (NGT); nevertheless, there was no significant difference in serum asprosin levels among subgroups both in acromegaly and controls (acromegaly: *F* ＝ 0.658, *P*=0.521, controls: *H* ＝ 0.045, *P*=0.978, Figure 2(c)). Considering the effect of diabetes treatment on serum asprosin levels, acromegaly patients with diabetes treatment (treatment details about DM in controls were unknown) were further excluded, and still there was no difference in serum asprosin levels among subgroups in acromegaly patients (*F* ＝ 1.211, *P*=0.546).

### 3.4. Bivariate Correlation Analysis of Serum Asprosin Levels with Other Variables

To investigate the correlation between serum asprosin levels and other variables, bivariate correlation analysis was used and results are shown in Table 3. In acromegaly patients, serum asprosin levels were negatively correlated with the course of acromegaly (*r* ＝ −0.273, *P*=0.026), IGF-1 SDS (*r* ＝ −0.295, *P*=0.016), GH-Nadir (*r* ＝ −0.249, *P*=0.046), GH-AUC (*r* ＝ −0.285, *P*=0.021), and Cr (*r* ＝ −0.415, *P*=0.001), while they were positively correlated with TC (*r* ＝ 0.614, *P* < 0.001) and LDL-C (*r* ＝ 0.560, *P* < 0.001). Serum asprosin levels in total subjects were also positively correlated with TC (*r* ＝ 0.184, *P*=0.025).

In acromegaly patients, serum asprosin levels were negatively correlated with gender (*r* ＝ −0.428, *P* < 0.001) and age (*r* ＝ −0.285, *P*=0.018), but not with blood glucose parameters (fasting blood glucose, FBG, and two-hour postprandial blood glucose, 2h-Glu). Also, serum asprosin levels in controls were negatively correlated with gender (*r* ＝ −0.565, *P* < 0.001) and age (*r* ＝ −0.260, *P*=0.005) and had no correlations with blood glucose parameters. Besides, the same results were observed in total subjects (gender: *r* ＝ −0.323, *P* < 0.001 and age: r ＝ −0.226, *P*=0.002).

In addition, serum asprosin levels were positively correlated with BMI (*r* ＝0.344, *P* < 0.001; *r* ＝ 0.270, *P* < 0.001) and HDL-C (*r* ＝ 0.315, *P*=0.001; *r* ＝ 0.333, *P* < 0.001) in controls and total subjects, but negatively correlated with UA (*r* ＝ −0.351, *P* < 0.001; *r* ＝ −0.270, *P* < 0.001).

### 3.5. Multiple Regression Analysis of Serum Asprosin Levels

Moving on to the associations between serum asprosin levels and other variables, multiple stepwise linear regression analysis was used. Multiple stepwise linear regression analysis (Table 4) revealed acromegaly was an independent influencing factor of serum asprosin levels. Serum asprosin levels were negatively correlated with acromegaly (*β* ＝ −0.273, *P* < 0.001, acromegaly assigned to 1, controls assigned to 0), which indicated that serum asprosin levels in acromegaly patients were lower than controls. Additionally, sex, age, BMI, and TC were independent influencing factors of serum asprosin levels. Serum asprosin levels were negatively correlated with gender (*β* = −0.578, *P* < 0.001, male assigned to 1, female assigned to 0) and age (*β* ＝ −0.193, *P*=0.004), while they were positively correlated with BMI (*β* ＝ 0.225, *P*=0.001) and TC (*β* ＝ 0.152, *P*=0.025).

### 3.6. Serum Asprosin Levels and the Risk of Acromegaly

In order to further explore serum asprosin levels of patients with acromegaly, patients with acromegaly and controls were stratified into lowest group, median group, and highest group according to serum asprosin tertiles levels (lowest: <2.12 ng/ml; median: 2.12 ∼ 3.06 ng/ml; highest: ≥3.06 ng/ml). As shown in Table 5, univariate binary logistic regression analysis showed that the risk of acromegaly in the lowest group was 2.67 times higher than the highest group (OR ＝ 3.665, 95% CI 1.677–8.007, *P*=0.001). With gender, age, and BMI being further adjusted in model 1, it was observed that the lowest group also has an increased risk of acromegaly with 6.26 times higher than highest group (OR ＝ 7.262, 95% CI 2.248 – 23.457, *P*=0.001). Furthermore, TC was further adjusted in model 2, and the increased risk of acromegaly in the lowest group still existed (OR ＝1 8.645, 95% CI 3.757–92.540, *P* < 0.001). Thus, patients with low serum asprosin levels had a higher risk of developing acromegaly compared with high serum asprosin levels, suggesting lower serum asprosin levels in patients with acromegaly.

## 4. Discussion

Our study was the first to demonstrate that serum asprosin levels in acromegaly patients were significantly lower than controls. At the same time, serum asprosin levels in patients with the acromegaly course ≥5 years, high IGF-1 SDS group, and high GH-AUC were significantly decreased. Negative correlations between serum asprosin levels and the course of acromegaly, IGF-1 SDS, GH-Nadir, and GH-AUC in acromegaly patients were also observed. Regardless of acromegaly, serum asprosin levels in women exceeded those in men. Furthermore, serum asprosin levels in acromegaly patients and total subjects were positively correlated with TC, but not BMI in acromegaly patients.

In this research, we preliminarily showed that serum asprosin levels in acromegaly patients were lower than controls. Meanwhile, serum asprosin levels in patients with the acromegaly course ≥5 years, high IGF-1 SDS, and high GH-AUC were significantly decreased. Besides, negative correlations between serum asprosin levels and the course of acromegaly, IGF-1 SDS, GH-Nadir, and GH-AUC in acromegaly patients were observed. The possible reasons for lower asprosin levels in acromegaly were as follows. Firstly, asprosin secretion is intimately related to blood glucose levels. Romere et al. [2] showed that plasma asprosin levels in mice immediately decreased with the increased blood glucose levels after feeding, while they increased with the decreasing blood glucose after fasting overnight, suggesting that blood glucose might be a regulatory factor to asprosin secretion. Interfering vitro cultured mouse hepatocytes with different concentrations of asprosin, release of glycogen increased with increasing asprosin concentrations. Further studies indicated that asprosin could activate the G protein-coupled receptor-cAMP-PKA pathway, induce rapid decomposition and release of liver glycogen, and increase blood glucose. Besides, Wiecek et al. [22] found that, within 30 minutes after anaerobic exercise, blood glucose levels of women gradually decreased, while serum asprosin levels gradually increased, demonstrating that decreased blood glucose may induce increased secretion of asprosin. It was found that patients with acromegaly did have higher fasting blood glucose than controls in this study, though both within normal range. Therefore, increased blood glucose in patients with acromegaly may inhibit asprosin secretion. Secondly, decreased adipose tissue by long-term GH exposure in acromegaly patients exerts a negative impact on asprosin synthesis and secretion. To our knowledge, the effect of GH on adipose tissue is to promote lipid mobilization and oxidation, and body fat composition of acromegaly patients after long-term growth hormone exposure has decreased [12]. Previous study of neurosurgery department in our center demonstrated that patients with acromegaly had lower total adipose tissue weight and visceral fat index (8.73 ± 2.19 vs 10.24 ± 2.45) than patients with nonfunctional pituitary tumors matched by gender, age, and BMI [23]. On the contrary, increased waist circumference, fat mass, and body fat rate of adult growth hormone deficient patients about 7 years after discontinuing growth hormone replacement were observed in our previous study [24]. Furthermore, our results found that acromegaly was an independent influential factor for serum asprosin levels, further suggesting that decreased serum asprosin levels in acromegaly patients were related to long-term high GH levels. As we all know, asprosin is mainly secreted by white adipose tissue; thus, we believe that lower serum asprosin levels in acromegaly patients may be associated with long-term effects of high GH levels. However, further studies are needed to explore how GH affects the synthesis and secretion of asprosin.

Serum asprosin levels in both acromegaly patients and controls showed sexually dimorphic and women were significantly higher than men. Further bivariate correlation analysis and multiple linear regression analysis also showed that serum asprosin levels were negatively correlated with gender (male 1, female 0), which indicated serum asprosin levels in female patients were higher. Importantly, gender is a vital factor in distribution of body adipose tissue. In general, women have a higher proportion of adipose tissue than men, and storages of adipose in female are mainly white adipose tissue in visceral gonad and subcutaneous in the groin, while mainly visceral gonad white adipose tissue in men [25]. Thus, higher percent of white adipose tissue in women has more asprosin synthesis and secretion, leading to higher serum asprosin levels. Moreover, more remarkable adipose reduction in acromegaly men may be associated with less asprosin synthesis and secretion. Several researches have demonstrated that decreased fat mass was more obvious in men under long-term GH exposure. Guo et al. [23] revealed that total fat tissue mass and visceral fat index were obviously decreased in acromegaly men with an average course of 7 years (89.3 months) compared with nonfunctional pituitary tumors patients matched by age, sex, and BMI, but no significant changes in women. Additionally, a prospective study by Götherström et al. [26] on adult growth hormone deficiency found that fat mass reduction in men after 5 years of growth hormone replacement was more significant than women. Therefore, regardless of acromegaly or not, serum asprosin levels in women are higher than men.

In addition, our study showed that serum asprosin levels in obesity were higher than normal in controls and consistent with previous studies on serum asprosin levels in obese patients [6, 9, 27], and serum asprosin levels were positively correlated with BMI in controls and total subjects in our study. However, there was no correlation between serum asprosin levels and BMI in acromegaly patients, and serum asprosin levels in different BMI groups had no significant difference. It is well known that BMI is a rough indicator to assess the ratio of weight to height and cannot objectively reflect body fat composition [26]. As mentioned above, patients with acromegaly have reduced fat mass and changed body fat composition due to high GH levels, which may lead to no correlation between serum asprosin levels and BMI.

Furthermore, serum asprosin levels in acromegaly patients and total subjects were positively correlated with TC, and multiple linear regression analysis showed that TC was an independent influencing factor of serum asprosin levels. To our knowledge, serum asprosin levels in metabolic diseases patients are correlated with lipid metabolism indexes, which support our results [11]. Serum asprosin levels in PCOS patients were positively correlated with TC (r ＝ 0.389, *P*=0.019) [10]. At the same time, serum asprosin levels in diabetic patients were positively correlated with TG, and TG was an independent influencing factor of serum asprosin levels in diabetic patients [8, 27]. Therefore, serum asprosin levels are associated with lipid metabolism indexes, but not with BMI in acromegaly patients, and its relationship with abnormal glycolipid metabolism in acromegaly needs further researches.

Our study preliminarily explored serum asprosin levels in patients with acromegaly and its correlation with high GH levels and glucolipid metabolism indexes. However, this study had some limitations as follows. Firstly, as an observational study, the causal relationship between serum asprosin levels and other variables remained unclear, and further mechanism researches were needed. Secondly, lacking information like body fat distribution, insulin sensitivity, and so on, it was hard to fully understand the decrease of serum asprosin levels in acromegaly patients. In addition, diabetes treatment details of 7 patients (though accounting for only a percent of 5.8) in controls were unknown, and its effect on serum asprosin levels could not be evaluated.

## 5. Conclusion

This study for the first time showed that serum asprosin levels in acromegaly patients were lower than controls and related to long-term high GH levels. In addition, our study enriches adipocytokines spectrum of acromegaly and reveals the relationship between serum asprosin and high GH levels. However, the association between serum asprosin levels and glucolipid metabolism in acromegaly patients requires further researches.

## Figures and Tables

**Table 1:** Basic characteristics of acromegaly patients and controls.

Variables	Acromegaly (*N* = 68)	Controls (*N* = 121)	*P*
M/F, %	29/39, 74% (68)	43/78, 55% (121)	0.334
Age (y)!	41.3 ± 15.0 (68)	44.5 ± 13.0 (121)	0.146
SBP (mmHg)	123.2 ± 14.5 (68)	125.1 ± 18.7 (121)	0.565
DBP (mmHg)	77.5 ± 11.9 (67)	79.1 ± 11.8 (121)	0.349
BMI (kg/cm^2^)	25.79 ± 3.52 (66)	26.44 ± 3.41 (121)	0.192
ALT (U/L)!	18.4 ± 9.7 (67)	22.4 ± 1.2 (120)	**0.027**
AST (U/L)!	18.2 ± 6.2 (44)	21.0 ± 7.6 (115)	**0.030**
Cr (*μ*mol/l)!	55.0 ± 12.3 (66)	78.1 ± 23.3 (120)	**<0.001**
UA (*μ*mol/l)	277 ± 77 (61)	274 ± 75 (114)	0.762
TC (mmol/l)	4.49 ± 0.85 (36)	4.81 ± 0.85 (119)	**0.033**
TG (mmol/l)!	1.79 ± 1.09 (36)	1.72 ± 1.37 (119)	0.328
HDL-C (mmol/l)!	1.19 ± 0.31 (36)	1.40 ± 0.36 (115)	**0.004**
LDL-C (mmol/l)	2.62 ± 0.74 (36)	2.93 ± 0.70 (113)	**0.013**
FBG (mmol/l)!	6.0 ± 1.7 (66)	5.4 ± 0.9 (119)	**0.003**
2h-Glu (mmol/l)	9.0 ± 3.9 (66)	8.0 ± 3.3 (66)	0.081
Glu-AUC (mmol/l)	1615.3 ± 552.1 (66)	NA	NA
Asprosin (ng/ml)!	2.18 ± 0.86 (68)	2.71 ± 0.86 (114)	**<0.001**
D-max (mm)	19.8 ± 9.0 (59)	—	—
Course (y)	5.0 (2.3, 10.0) (68)	—	—
IGF-1 SDS	6.56 ± 2.43 (66)	—	—
GH (ng/ml)	15.0 (7.2, 32.6) (66)	—	—
GH-nadir (ng/ml)	10.7 (5.1, 24.0) (65)	—	—
GH-AUC (ng/ml)	2391.0 (1345.0, 5673.0) (65)	—	—

Values are expressed as mean ± standard deviation or median (P25, P75). !, non-parametric test. SBP: systolic blood pressure; DBP: diastolic blood pressure; FBG: fasting blood glucose; 2 h-Glu: 2-hour postprandial blood glucose; GLU-AUC: glucose of the area under curve of 75 g OGTT; D-max: maximum of tumor diameter; Course: course of acromegaly; IGF-1 SDS : SDS of insulin-like growth factor; GH-Nadir: nadir GH level after 75 g OGTT; GH-AUC: growth hormone of the area under curve of 75 g OGTT; NA, not available.

Bold *P* values indicate statistical significance (*P* < 0.05).

**Table 2:** Proportions of metabolic diseases in acromegaly patients.

Metabolic diseases	Acromegaly	Controls	*P*
	N/total (%)	N/total (%)	
*Glucose metabolism*			
Diabetes (DM)	19/66 (28.8)	7/66 (10.6)	0.009
Impaired glucose tolerance (IGT)	17/66 (25.8)	24/66 (36.4)	0.188
Normal	30/66 (45.5)	35/66 (53.0)	0.384

*BMI*			
Obesity	19/66 (28.8)	36/121 (29.8)	0.890
Overweight	27/66 (40.9)	53/121 (43.8)	0.702
Normal	20/66 (30.3)	32/121 (26.4)	0.574
Hypertension	17/68 (25.0)	29/121 (24.0)	0.874
Hyperlipidemia	20/38 (52.6)	58/120 (48.3)	0.644

**Table 3:** Bivariate correlation analysis of serum asprosin levels.

Variables	Acromegaly		Controls		Total	
	*r*	*P*	*r*	*P*	*r*	*P*
Gender	−0.428	<**0.001**	−0.565	<**0.001**	−0.323	<**0.001**
Age (y)	−0.285	**0.018**	−0.260	**0.005**	−0.226	**0.002**
SBP (mm Hg)	0.069	0.576	−0.095	0.315	−0.36	0.630
DBP (mm Hg)	0.098	0.431	−0.034	0.717	0.019	0.802
BMI (kg/cm^2^)	0.077	0.538	0.344	<**0.001**	0.270	<**0.001**
ALT (U/L)	−0.186	0.131	−0.028	0.769	−0.032	0.666
AST (U/L)	−0.038	0.808	−0.099	0.306	−0.029	0.725
Cr (*μ*mol/l)	−0.415	**0.001**	−0.049	0.604	−0.001	0.991
UA (*μ*mol/l)	−0.172	0.186	−0.351	<**0.001**	−0.270	<**0.001**
TC (mmol/l)	0.614	<**0.001**	0.003	0.978	0.184	**0.025**
TG (mmol/l)	0.292	0.084	0.028	0.769	0.069	0.403
HDL-C (mmol/l)	0.198	0.247	0.315	**0.001**	0.333	<**0.001**
LDL-C (mmol/l)	0.560	<**0.001**	−0.130	0.182	0.088	0.294
FBG (mmol/l)	−0.220	0.075	0.072	0.448	−0.091	0.223
2h-Glu (mmol/l)	−0.053	0.672	0.106	0.397	−0.022	0.807
Glu-AUC (mmol/l)	−0.147	0.237	—	—	—	—
D-max (mm)	0.015	0.910	—	—	—	—
Course (y)	−0.273	**0.026**	—	—	—	—
IGF-1 SDS	−0.295	**0.016**	—	—	—	—
GH (ng/ml)	−0.215	0.083	—	—	—	—
GH-Nadir (ng/ml)	−0.249	**0.046**	—	—	—	—
GH-AUC (ng/ml)	−0.285	**0.021**	—	—	—	—

SBP: systolic blood pressure; DBP: diastolic blood pressure; FBG: fasting blood glucose; 2 h-Glu: 2-hour postprandial blood glucose; GLU-AUC: glucose of area under curve of 75 g OGTT test; D-max: maximum of tumor diameter; Course: course of acromegaly; IGF-1 SDS: SDS of insulin-like growth factor; GH-Nadir: nadir GH level after 75 g OGTT; GH-AUC: growth hormone of the area under curve after 75 g OGTT; NA, not available. Bold *P* values indicate statistical significance (*P* < 0.05).

**Table 4:** Multiple regression analysis of variables independently related to serum asprosin in all subjects.

Asprosin^#^ (R^2^adj = 0.626)	Multiple stepwise linear regression			
	Unstandardized coefficients (B)	Standard error	Standardized coefficients (*β*)	*P*
Constant	0.702	0.418		
Sex (male 1, female 0)	−0.560	0.063	−0.578	<**0.001**
Age^#^	−0.237	0.081	−0.193	**0.004**
BMI^#^	0.028	0.008	0.225	**0.001** ^*∗∗*^
Group (acromegaly 1, control 0)	−0.246	0.059	−0.273	<**0.001**
TC^#^	0.380	0.167	0.152	**0.025**

^#^ln-converted before analysis. Bold *P* values indicate statistical significance (*P* < 0.05).

**Table 5:** Binary logistic regression analysis of acromegaly risk according to asprosin tertiles levels.

Measurement	Tertiles		
	Lowest OR (95%CI)	Median OR (95%CI)	Highest OR (95%CI)
Range (ng/ml)	≤2.12	<2.12 to <3.06	≥3.06
Acromegaly/controls	31/29	23/37	14/48
Univariate	3.665 (1.677 ∼ 8.007)	2.131 (0.967 ∼ 4.699)	1.00 (reference)
*P*	**0.001**	0.061	
Model 1	7.262 (2.248 ∼ 23.457)	2.786 (1.152 ∼ 6.738)	1.00 (reference)
*P*	**0.001**	**0.023**	
Model 2	18.645 (3.757 ∼ 92.540)	3.146 (0.976 ∼ 10.137)	1.00 (reference)
*P*	<**0.001**	0.055	

Model 1: adjusted sex, age, and BMI. Model 2: further adjusted TC based on model 1.

## Data Availability

The data used to support the findings of this study are available from the corresponding author upon request.

## References

[1] Kojta I., Chacińska M., Błachnio-Zabielska A. (2020). Obesity, bioactive lipids, and adipose tissue inflammation in insulin resistance. *Nutrients*.

[2] Romere C., Duerrschmid C., Bournat J. (2016). Asprosin, a fasting-induced glucogenic protein hormone. *Cell*.

[3] Lee T., Yun S., Jeong J. H., Jung T. W. (2019). Asprosin impairs insulin secretion in response to glucose and viability through TLR4/JNK-mediated inflammation. *Molecular and Cellular Endocrinology*.

[4] Jung T. W., Kim H. C., Kim H. U. (2019). Asprosin attenuates insulin signaling pathway through PKC*δ*‐activated ER stress and inflammation in skeletal muscle. *Journal of Cellular Physiology*.

[5] Li E., Shan H., Chen L. (2019). OLFR734 mediates glucose metabolism as a receptor of asprosin. *Cell Metabolism*.

[6] Duerrschmid C., He Y., Wang C. (2017). Asprosin is a centrally acting orexigenic hormone. *Nature Medicine*.

[7] Liu Y., Long A., Chen L., Jia L., Wang Y. (2020). The Asprosin-OLFR734 module regulates appetitive behaviors. *Cell Discovery*.

[8] Zhang L., Chen C., Zhou N., Fu Y., Cheng X. (2020). Circulating asprosin concentrations are increased in type 2 diabetes mellitus and independently associated with fasting glucose and triglyceride. *Clinica chimica acta*.

[9] Wang C.-Y., Lin T.-A., Liu K.-H. (2019). Serum asprosin levels and bariatric surgery outcomes in obese adults. *International Journal of Obesity*.

[10] Li X., Liao M., Shen R. (2018). Plasma asprosin levels are associated with glucose metabolism, lipid, and sex hormone profiles in females with metabolic-related diseases. *Mediators of Inflammation*.

[11] Yuan M., Li W., Zhu Y., Yu B., Wu J. (2020). Asprosin: a novel player in metabolic diseases. *Frontiers in Endocrinology*.

[12] Olarescu N. C., Bollerslev J. (2016). The impact of adipose tissue on insulin resistance in acromegaly. *Trends in Endocrinology & Metabolism*.

[13] Silha J. V., Krsek M., Hana V. (2003). Perturbations in adiponectin, leptin and resistin levels in acromegaly: lack of correlation with insulin resistance. *Clinical Endocrinology*.

[14] Bolanowski M., Milewicz A., Bidzińska B. (2002). Serum leptin levels in acromegaly--a significant role for adipose tissue and fasting insulin/glucose ratio. *Medical Science Monitor: International Medical Journal of Experimental and Clinical Research*.

[15] Fukuda I., Hizuka N., Ishikawa Y. (2004). Serum adiponectin levels in adult growth hormone deficiency and acromegaly. *Growth Hormone & IGF Research*.

[16] Ronchi C., Corbetta S., Cappiello V. (2004). Circulating adiponectin levels and cardiovascular risk factors in acromegalic patients. *European Journal of Endocrinology*.

[17] Berryman D., List E. (2017). Growth hormone’s effect on adipose tissue: quality versus quantity. *International Journal of Molecular Sciences*.

[18] Katznelson L., Laws E. R., Melmed S. (2014). Acromegaly: an endocrine society clinical practice guideline. *The Journal of Clinical Endocrinology & Metabolism*.

[19] Chinese joint committee on the revision of guidelines for the prevention and treatment of dyslipidemia in adults (2016). Chinese guidelines for prevention and treatment of dyslipidemia in adults (2016 revision). *Chinese Circulation Journal*.

[20] Chinese Medical Association Endocrinology Branch Obesity Group (2011). Chinese experts consensus on prevention and tretment of obesity. *Chinese Journal of Endocrinology and Metabolism*.

[21] Unger T., Borghi C., Charchar F. (2020). 2020 International Society of Hypertension global hypertension practice guidelines. *Journal of Hypertension*.

[22] Wiecek M., Szymura J., Maciejczyk M., Kantorowicz M., Szygula Z. (2018). Acute anaerobic exercise affects the secretion of asprosin, irisin, and other cytokines-a comparison between sexes. *Frontiers in Physiology*.

[23] Guo X., Gao L., Shi X. (2018). Pre- and postoperative body composition and metabolic characteristics in patients with acromegaly: a prospective study. *International Journal of Endocrinology*.

[24] Yang H., Wang L., Qiu X. (2018). Body composition and metabolic health of young male adults with childhood-onset multiple pituitary hormone deficiency after cessation of growth hormone treatment. *Journal of Pediatric Endocrinology and Metabolism*.

[25] Varghese M., Griffin C., Singer K. (2017). The role of sex and sex hormones in regulating obesity-induced inflammation. *Sex and Gender Factors Affecting Metabolic Homeostasis, Diabetes and Obesity*.

[26] Götherström G., Svensson J., Koranyi J. (2001). A prospective study of 5 years of GH replacement therapy in GH-deficient adults: sustained effects on body composition, bone mass, and metabolic indices. *The Journal of Clinical Endocrinology & Metabolism*.

[27] Wang Y., Qu H., Xiong X. (2018). Plasma asprosin concentrations are increased in individuals with glucose dysregulation and correlated with insulin resistance and first-phase insulin secretion. *Mediators of Inflammation*.

